# Evaluation of a constant rate intravenous infusion of dexmedetomidine on the duration of a femoral and sciatic nerve block using lidocaine in dogs

**DOI:** 10.3389/fvets.2022.1061605

**Published:** 2023-01-13

**Authors:** Marzia Stabile, Luca Lacitignola, Claudia Acquafredda, Annalaura Scardia, Antonio Crovace, Francesco Staffieri

**Affiliations:** Section of Veterinary Clinics and Animal Production, Department of Emergency and Organ Transplantation, University of Bari “Aldo Moro”, Bari, Italy

**Keywords:** adjuvant, block duration, dexmedetomidine, dog, lidocaine, sensory blockade

## Abstract

**Objectives:**

This study investigated the effects of 1 μg/kg/h intravenous constant rate infusion (CRI) of dexmedetomidine on the sensory and motor blockade for femoral and sciatic nerve blocks in dogs undergoing stifle surgery.

**Materials and methods:**

Client-owned dogs referred for stifle surgery were enrolled in this prospective, randomized, blinded study. Dogs were pre-medicated with acepromazine (0.005–0.01 mg/kg intramuscularly, IM); anesthesia was induced with propofol intravenously and maintained with isoflurane in a mixture of air and oxygen. Electrolocation-guided sciatic and femoral nerve blocks with lidocaine 2% (0.15 mL/kg) were performed using the parasacral and lateral pre-iliac approaches, respectively. After performing local block, a systemic infusion of saline solution (group C) or dexmedetomidine (group D) was started at a CRI at 1 ml/kg/h and continued until the end of surgery. Dexmedetomidine was infused at a dose of 1 μg/kg/h. Respiratory and hemodynamic variables were recorded during surgery. Sensory and motor blockade was evaluated by response to pinching the skin innervated by the sciatic/femoral nerves, with forceps and by observing the dogs' ability to walk and testing proprioception at 30, 60, 120, 180, and 240 min after extubation. Analgesia was monitored with SF-GCPS. Methadone IM was administered as rescue analgesia. Intraoperative data were analyzed by analysis of variance, while postoperative data were analyzed by the independent two-tailed *t*-test and a Kaplan–Meier test (*p* < 0.05).

**Results:**

Twenty dogs were included in this study (10/group). A significant difference in the recovery of sensory nerve function was observed between the groups. The mean durations of the sensory blockade for femoral and sciatic nerves, respectively, was longer (*p* < 0.001) for group D [168 (146–191, 95% CI), 161 (143–179, 95% CI) min] than in group C [120 (96.1–144, 95% CI), 116 (90.9–142, 95% CI]. No differences in the recovery of patellar and tibial reflexes, proprioceptive function, and ability to walk were found among groups. The overall postoperative rescue analgesia requirement was significantly different (*p* = 0.019) between groups, with an incidence of 5/10 (50%) dogs in group D and 10/10 (100%) dogs in group C.

**Conclusion:**

Dexmedetomidine administered as a CRI (1 μg/kg/h) combined with local lidocaine increases the duration of the sensory component of the sciatic and femoral nerve blocks and reduces the requirement for additional analgesia during the immediate postoperative hours.

## 1. Introduction

Regional anesthesia (RA) is widely recognized as being a crucial element of balanced anesthesia. In a multimodal analgesic regimen, the use of RA is essential to assure an anesthetic-sparing effect, reduce perioperative opioid consumption and improve clinical recovery (1–3). Local anesthetics (LAs) are classified based on their solubility, onset time, duration, and toxicity (4, 5). Among the LAs most used in veterinary medicine, lidocaine is the only one licensed for use in animals, with excellent solubility, fast onset, short duration of action (~1 h), and few side effects (6).

Because of lidocaine's short duration of action, many local anesthtic adjuvants have been studied as strategies for extending the duration of action. Adjuvants and LAs have historically been used in synergy to improve RA in different ways, including speeding up the onset, extending the duration, and enhancing the efficacy of the nerve block (7, 8). The main goals of their use should be to prolong the duration of the sensory blockade and reduce the dose of the LA, with a consequent decrease in potential side effects. Adjuvants can be administered in numerous ways including topically, perineurally, neuraxially, or systemically (7).

Dexmedetomidine, the d-isomer of medetomidine, is an alpha2-adrenoceptor agonist, normally used in clinical practice because of its sedative and analgesic effects in dogs and cats (9). For its analgesic properties, dexmedetomidine has been proposed as a local anesthetic adjuvant to RA in both human and veterinary medicine, in association with different LAs (10–16). In a recent clinical study in dogs, the adjuvant action of dexmedetomidine, at 0.15 μg/kg, administered with lidocaine prolonged the sensory blockade in the sciatic and femoral nerves by ~2.5 times compared to lidocaine alone (16). In contrast, dexmedetomidine did not prolong the effect with longer duration LAs such as bupivacaine and ropivacaine (14, 17).

The intravenous route for administering dexmedetomidine has been suggested to be similar to the perineural route in terms of adjuvants for LAs. In dogs, Acquafredda et al. proved that after intramuscular administration of 0.15 μg/kg of dexmedetomidine contemporary to the local block with lidocaine, the sensory blockade was prolonged by a factor of 1.5 (16). Furthermore, Sarotti et al. proved that the administration of dexmedetomidine as a constant rate infusion (CRI) intravenously in dogs prolonged the duration of the spinal block with lidocaine without affecting the duration of the motor block (12).

To the best of our knowledge, the effects of the intravenous administration of dexmedetomidine as a CRI combined with perineural administration of lidocaine for peripheral nerve block in dogs have not been reported. Therefore, this study aimed to investigate the effects of intravenously administered dexmedetomidine as a CRI concomitant with perineural lidocaine on the sensory and motor blockade of femoral and sciatic nerve blocks in dogs undergoing stifle surgery. We hypothesized that the systemic administration of dexmedetomidine would prolong the sensory blockade and improve postoperative analgesia, reducing postoperative rescue analgesia, when compared with lidocaine alone.

## 2. Materials and methods

### 2.1. Ethics statements

This study was approved by the Ethical Committee for Veterinary Clinical and Zootechnical Studies of the Department of Emergency and Organ Transplantation (D.E.O.T.) at the University of Bari, Italy (certificate of approval number: 02/2021). Informed consent was obtained from all the owners of the dogs before they were included in the study. This study followed the Consolidated Standards of Reporting Studies Statement 2010 for presenting randomized clinical trials (18).

### 2.2. Animals

We investigated client-owned dogs that presented to the veterinary surgery hospital of D.E.O.T. of the University of Bari between March 2021 and April 2022. Dogs were referred for surgical resolution of acute cranial cruciate ligament rupture using the tibial tuberosity advancement (TTA) technique.

Only dogs with an American Society of Anesthesiologists physical status < 3, those older than 6 months of age, and those with a body weight >10 kg were enrolled. A few days before the scheduled surgery, all dogs were clinically assessed, and blood tests were performed. Dogs diagnosed with systemic diseases (cardiac, hepatic, renal, or metabolic), those that were pregnant, or dogs who were extremely aggressive were excluded. Dogs with any condition that would preclude the administration of a peripheral nerve block, such as coagulopathies, skin infections, anatomical anomalies, or a history of negative reactions to LAs, were also excluded. Patients who underwent TTA surgery for >3 h were not included in the analysis.

Animals were housed in the hospital on the same day as the surgery, fasted overnight, and had *ad libitum* access to water until pre-anesthetic medication.

### 2.3. Study design

This investigation was planned as a prospective, blind, and randomized clinical trial. A computer-generated randomization process determined the allocation of dogs into one of two treatment groups. The entire study was conducted by the same operators (MS or CA), who were blinded to the allocation group of the animals. The same orthopedic surgeon (AC) performed each procedure.

### 2.4. Anesthesia protocol

Dogs were premedicated with acepromazine (5–10 μg/kg; Prequillan 1%; Fatro, Italy), injected intramuscularly (IM) into the left quadriceps muscle. A catheter was inserted intravenously (IV) into the cephalic vein 20 min after the administration of acepromazine to provide medications and fluids at a rate of 5 mL/kg/h (Ringer Lattato; Fresenius Kabi Italia Srl, Isola della Scala VR, Italy) during the perioperative period. Propofol (10 mg/mL; Fresenius Kabi Italia Srl) was injected IV to induce general anesthesia until orotracheal intubation could be performed. Isoflurane (Vet-Flurane, Virbac, Italy) was used to maintain anesthesia and was delivered using a rebreathing system in a mixture of oxygen and air (fraction of inspired oxygen = 0.5; Intersurgical Srl, Mirandola MO, Italy). Appropriate prophylactic antibiotics were administered to all dogs prior to surgery, and radiographs of the limbs were obtained when required for planning surgical intervention.

All dogs were mechanically ventilated with a volume-controlled mode ventilator (AX900 Comen, Foschi Srl, Roma R,M Italy). The tidal volume was set to 15 mL/kg, inspiratory-to-expiratory ratio was 1:2, inspiratory pause was 20% of the inspiratory time, and respiratory rate was changed in accordance with the end-tidal carbon dioxide (EtCO_2_, mmHg). The EtCO_2_ was maintained within a range of 35–45 mmHg. End-expiratory isoflurane fraction (EtIso, %) was kept constant for all dogs between 1.1 and 1.2% throughout the procedure.

A nerve stimulator (Plexygon Nerve Stimulator; Vygon Italia Srl, Padova PD, Italy) was used to guide perineural administration *via* a 22-gauge, 50–70-mm insulated needle with 30° cutting bevel and an exposed tip (Locoplex; Vygon Italia Srl). Muscle contraction induced by a 0.2–0.4-mA current was considered acceptable, and 0.15 mL/kg (19) of lidocaine (lidocaine 2%, Ecuphar Italia Srl, Milano MI, Italy) was slowly injected at each site.

After establishing a steady plane of anesthesia, sciatic and femoral nerve blocks were performed on the affected limb using the parasacral approach and lateral pre-iliac technique, as described by Portela et al. (20, 21).

Isoflurane was stopped at the end of the surgery, and the dogs were extubated once their swallowing reflex was restored. The length of the surgical procedure was calculated from the beginning of the first incision to the end of the last suture. The time of extubation, first head movements, sternal recumbency, and standing position following isoflurane discontinuation were also recorded for each dog.

### 2.5. Study protocol

The study protocol investigated the randomly assigned systemic infusion of saline solution alone (group C) or dexmedetomidine (group D; http://www.random.org; Randomness and Integrity Service Ltd., Dublin, Ireland). The solutions were prepared by an operator who was not involved in case management, as follows.

Group D: 0.1 mL of dexmedetomidine (Dexdomitor 0.5 mg/mL; Vetoquinol, Lure cedex, France) was added to 49.9 mL of saline solution (NaCl 0.9%, Fresenius Kabi Italia Srl) to obtain a final concentration of 1 μg/mL of dexmedetomidine.

Group C: 50 mL of saline solution (0.9% NaCl; Fresenius Kabi Italia Srl) was used.

The infusions started immediately after the execution of the local block and at least 10 min before the skin incision at a constant rate of 1 mL/kg/h (Syringe Pump SP3 Vet, Foschi Srl,), and was continued until the end of surgery, when the last stitch was placed and the vaporizer was turned off. Based on this, dexmedetomidine was infused at a dose of 1 μg/kg/h without a bolus.

### 2.6. Intraoperative assessment

Intraoperative monitoring was performed using a multi-parameter monitor (S/5 Compact; Datex-Ohmeda Oy, Helsinki, Finland) and included measuring the following variables every 5 min: heart rate (HR, beats per minute), respiratory rate (RR, breaths per minute), oxygen saturation (SpO_2_, percent), body temperature (T, °C), EtCO_2_ (mmHg), and isoflurane (EtIso, percent); and systolic arterial pressure (SAP, mmHg), diastolic arterial pressure (DAP, mmHg), and mean non-invasive arterial pressure (MAP, mmHg). Blood pressure measurements were collected using an oscillometric technique by placing the cuff (40% of limb circumference) on the dog's right or left antebrachium. During the intraoperative phase, specific time points were identified to evaluate nociceptive autonomic responses to surgical stimulation and confirm the success of the blocks. At baseline (1 min prior to skin incision), skin incision, bone manipulation, drilling, and final skin suturing, the HR, RR, MAP, SpO_2_, EtCO_2_, and T were recorded.

### 2.7. Event definition and treatment

An increase in HR, RR, or MAP by >20% from baseline during the surgical procedure was considered as nociception. Rescue analgesia with 1 μg/kg of fentanyl was administered IV. A second dose (1 μg/kg) was administered IV if the variables did not return within 20% of the baseline values 5 min after the initial dose. The block was judged unsuccessful if >3 boluses were necessary. In these cases, dogs were immediately removed from the study, and fentanyl infusion was started at a constant rate (5–10 μg/ kg/h).

An MAP < 60 mmHg for two consecutive measurements at a 3-min interval was considered hypotension. To treat this condition, the first action was to reduce the inhalant anesthetic dose, if possible, and then to administer 3 mL/kg of crystalloids over 5 min (Ringer's lactate); in cases of non-responsiveness, dopamine (3–7 μg/kg/min) was infused. An HR < 40 beats per minute for >1 min was defined as bradycardia and was treated with atropine (20 μg/kg IV), the systemic infusion (dexmedetomidine or saline solution) stopped, and the case was excluded from the study.

### 2.8. Postoperative assessment

During the postoperative phase, all dogs were monitored by the same trained blinded operator at 30, 60, 120, 180, and 240 min after extubation. At each time point, physiological parameters, pain, and the motor and sensory blockade were assessed.

The HR, RR, and T during recovery were measured clinically. Pain was assessed using the Italian version of the Short Form of the Glasgow Composite Pain Scale (SF-GCPS) (22). For a score >5 of 20 or 6 of 24 (based on the dog's ability to walk), 0.2 mg/kg of intravenous methadone (Semfortan 10 mg/mL; Dechra Pharmaceuticals, Northwich, UK), and 1 mg/kg of subcutaneous robenacoxib (Elanco Srl, Sesto Fiorentino FI, Italy) were administered to the dog. Thirty minutes after drug administration, pain was reassessed to confirm the treatment efficacy. If required, an additional dose of methadone (0.1 mg/kg) was administered intramuscularly. Pain management of dogs that received rescue analgesia continued based on the clinical needs until the end of the study. Animals were not withdrawn from the study after rescue analgesia and continued to be assessed as per the protocol. The time between extubation and the first administration of analgesics was calculated and recorded.

### 2.9. Sensory and motor blockade

The protocol used to test the sensory and motor blocks was the same as that used in previous studies (14, 16, 20). In particular, to test the sensory branch of the femoral and saphenous nerves, the skin of the medial aspect of the thigh was exposed to noxious stimuli using a Kelly clamp (Bontempi Srl, Cellatica BS, Italy). To test the sensory branch of the sciatic nerve (tibial and common fibular nerves), the skin over the caudal aspect of the metatarsus and over the third phalanx of the fourth digit was also stimulated. To prevent tissue trauma, the clamp jaws were covered with a few layers of surgical tape (Micropore Surgical tape; 3M Srl, Milan, Italy). Responses to these stimulations were graded as follows: 1 (no effect; normal response), 2 (attenuated response), or 3 (complete block; absence of a response). The period between perineural injection of the LA and the first return of scores of 1 or 2 after noxious stimulus was defined as the duration of sensory blockade. To complete the evaluation of sensory return, reactions to light stifle palpation were assessed and scored as absent (no reaction) or present (mild or normal painful reaction). The time between the perineural drug injection and reaction to joint palpation was recorded.

The proprioceptive response and walking ability were tested to determine the degree of motor blockade. Based on the evaluation of the alignment of the operated limb during walking and paw dorsiflexion, a proprioceptive response was evoked. Responses were graded as follows: 1 (no effect; normal motor response), 2 (partial loss; delayed response and altered limb alignment), or 3 (total loss; absence of a response and altered limb orientation while walking).

Walking ability was evaluated by observing the dog walking in a straight line on a non-slippery surface. A score of 1 (normal; no change in movement) or 2 (abnormal movement of the treated limb) was assigned. The length of motor blockade was defined as the time between perineural drug administration and the first return of scores of 1 or 2 for the proprioceptive examination and a score of 1 for ability to walk.

At the end of the 4 h of observation, all dogs received 1 mg/kg of subcutaneous robenacoxib (if not required as rescue) and were discharged from the hospital.

### 2.10. Statistical analysis

Statistical analysis was performed using commercially available software (23). The sample size was estimated using data from Acquafredda et al. (16). Accepting an alpha risk of 0.05 and a beta risk of 0.1 in a two-sided test, 10 dogs in each group were necessary to recognize a statistically significant a difference ≥30 min, anticipating a dropout rate of 5% (www.granmo.es).

Parametric data were tested for normality using the Shapiro–Wilk test and are summarized as mean ± standard deviation and mean with 95% confidential interval (CI). Differences between groups were tested using an independent two-tailed *t*-test or Mann–Whitney *U*-test according to distribution. Analysis of variance was used to analyze the intraoperative parameters between the groups at different times. Kaplan–Meier curves were used to describe the percentage of rescue analgesia required at different times in the postoperative period. The log-rank test was used to capture the differences between curves. Statistical significance was set at *p* < 0.05.

## 3. Results

**Figure 1:**
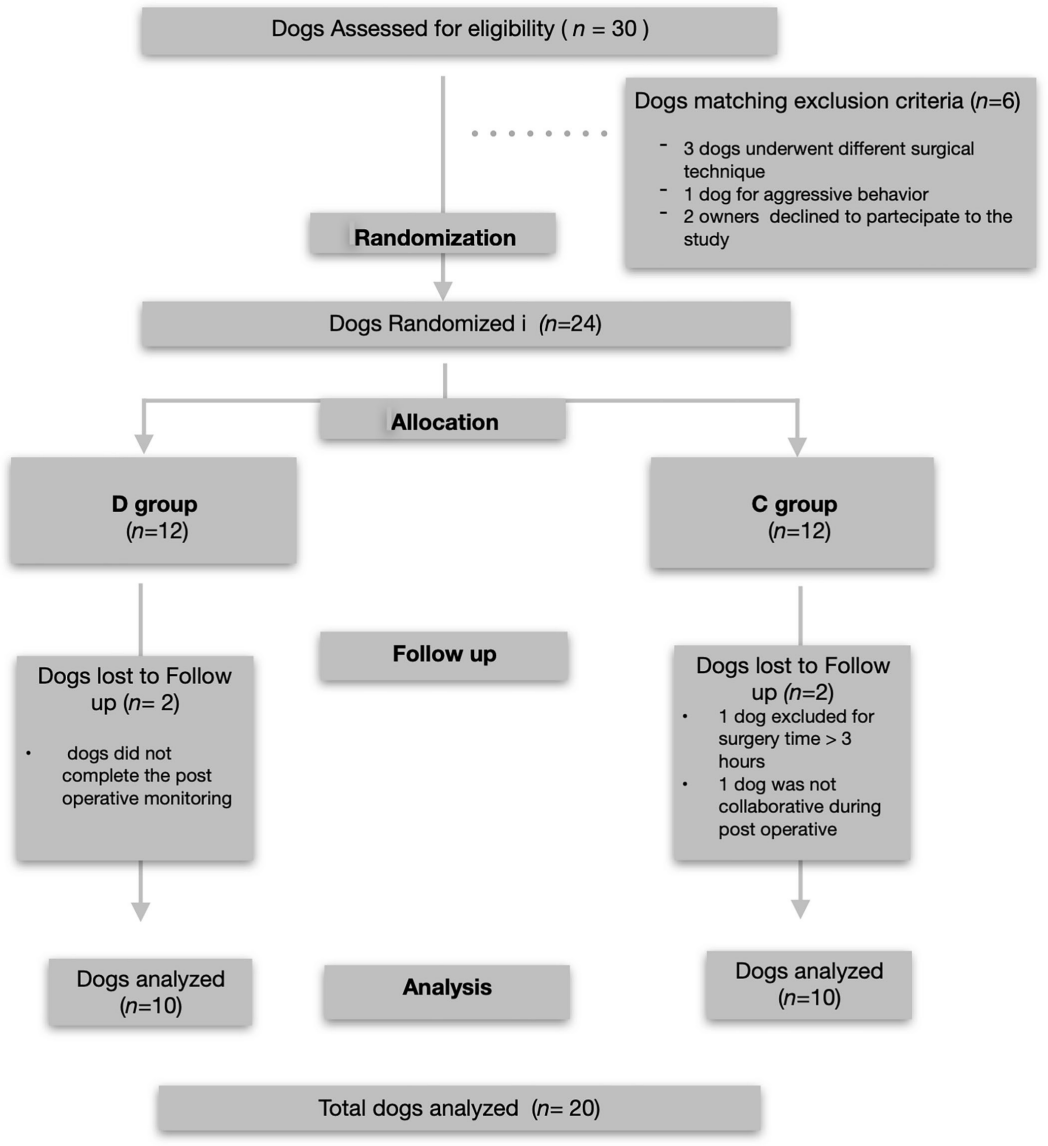
Consolidated Standards of Reporting Studies Statement flow diagram of the enrollment and group allocation.

Thirty dogs were initially recruited and underwent orthopedic stifle surgery. Six of these dogs did not meet the inclusion criteria; therefore, 24 dogs (12/treatment group) were included in the study. Four dogs (2/group) were excluded from the analysis because they did not complete the entire postoperative assessment. Data from 20 dogs (10 per group; 13 female and seven male dogs) who completed the study without complications were analyzed (Figure 1).

No statistical differences were found between the groups in terms of sex, age, weight, body condition score, surgery time, anesthesia time, and infusion time (Table 1).

**Table 1:** Demographic data and surgery data.

**Characteristic**	**Group D**	**Group C**	***p*-value**
Sex (male/female)	3/7	4/6	
Age (year)	7 ± 3	6.8 ± 2.1	0.86
Weight (kg)	28.4 ± 10.9	29.0 ± 6.8	0.89
BCS (1–9)	5.5 (5–8)	6 (5–9)	0.64
Surgery time (min)	42.6 ± 10.4	35.7 ± 9.9	0.14
Anesthesia time (min)	113 ± 17.1	121 ± 12.8	0.25
Infusion time (min)	54.3 ± 10.6	50.0 ± 9.1	0.34

Data are presented as mean ± standard deviation, median and range (BCS), or number (sex); n = 10 for all groups.

BCS, body condition score.

In addition, no difference was found in physiological parameters recorded at specific time points during anesthesia compared to baseline values in both groups. The RR, T, SpO_2_, and EtCO_2_ values were not significantly different between the groups. However, the HR was significantly lower in group D than in group C (*p* = 0.001) at each time point, and MAPs were higher at baseline, skin incision, and skin suture in group D than in group C (*p* = 0.02; Table 2).

**Table 2:** Physiological variables recorded at predetermined times during surgery.

	**Group**	**Baseline**	**Skin incision**	**Bone manipulation**	**Bone drilling**	**Skin suture**
HR (beats per min)	D	78 ± 17^*^	77 ± 22^*^	81 ± 22^*^	75 ± 16^*^	69 ± 17^*^
	C	112 ± 16	114 ± 17	115 ± 17	112 ± 18	111 ± 18
RR (breaths per min)	D	12 ± 2	12 ± 3	12.5 ± 2	12 ± 1	12 ± 1
	C	13 ± 5	13 ± 4	13.5 ± 5	13 ± 5	13 ± 4
MAP (mmHg)	D	85 ± 9^*^	87 ± 11^*^	92 ± 16	89 ± 14	93 ± 16
	C	73 ± 15	77 ± 14	85 ± 13	84 ± 12	78 ± 16
SpO_2_ (%)	D	98 ± 1	98 ± 1	99 ± 1	99 ± 1	99 ± 1
	C	98 ± 1	98 ± 0.5	98 ± 1	99 ± 1	98 ± 2
EtCO_2_ (mmHg)	D	36 (35–37.8)	38 (37–40.5)	39 (36–42.8)	37 (35.3–41.8)	38 (36–41.5)
	C	37 (35.3–40)	38 (36.3–41.5)	36 (35–40.8)	36 (36–40.8)	37 (36–40.8)
T (°C)	D	37.4 ± 1.8	37.3 ± 1.8	37.1 ± 2.2	37 ± 2.2	36.8 ± 2.9
	C	36.7 ± 3	36.5 ± 3.1	36.6 ± 4.1	36.8 ± 1.8	36.1 ± 2.8

Data are presented as mean ± standard deviation or median and range (EtCO_2_).

^*^p-value < 0.05 was considered statistically significant compared to the group C.

HR, heart rate; RR, respiratory rate; MAP, mean arterial pressure; SpO_2_, oxygen saturation; EtCO_2_, end-tidal carbon dioxide; T, temperature.

In each group, 1 dog experienced a single event of hypotension that was resolved by reducing the exhaled inhalant anesthetic and administering a crystalloid bolus (3 mL/kg) within 5 min. Bradycardia was not observed in any of the cases. In two cases in group D and three cases in group C, intraoperative rescue analgesia was required as a single bolus of fentanyl (1 μg/kg) during surgery. These events did not affect the study of these patients, and the local block was judged successful. All dogs recovered well without complications from anesthesia. Times to extubation and sternal recumbency were longer in group D than in group C (12 ± 6 vs. 5 ± 2 min, *p* = 0.001 and 35 ± 18 vs. 18 ± 10 min, *p* = 0.03, respectively). There was no difference in the time of the first head movement and standing between the groups (Table 3).

**Table 3:** Times of recovery between the groups.

	**Group D**	**Group C**	***p*-value**
Time from end of anesthesia to extubation (min)	12 ± 6	5 ± 2	0.001^*^
Time from end of anesthesia to first head movement (min)	20 ± 11	15 ± 10	0.2
Time from end of anesthesia to sternal recumbency (min)	35 ± 18	18 ± 10	0.03^*^
Time from end of anesthesia to standing	98 ± 59	85 ± 30	0.5

Data are presented as mean ± standard deviation.

^*^A *p*-value < 0.05 was considered statistically significant compared to group C.

A significant difference in the recovery of sensory nerve function was observed between the groups. The mean duration of the sensory blockade for femoral and saphenous, tibial and common fibular nerves was longer (*p* < 0.001) in group D (168, 95% CI: 146–191 and 161, 95% CI: 143–179 min, respectively) than in group C (120, 95% CI: 96.1–144 and 116, 95% CI: 90.9–142 min, respectively) (Table 4). No differences in the recovery of patellar and tibial reflexes, proprioceptive function (score 1–2) and ability to walk (score 1) were found between the groups. The results related to the sensory and motor blockade are presented in Table 4. Data related to the HR, RR, and T measured clinically during recovery are shown in Table 5.

**Table 4:** Durations of sensory and motor blockades.

**Outcome**		**Group D**	**Group C**	***p*-value**
Duration of sensory blockade (min)	Sciatic nerve sensitivity	161 (143–179)	116 (90.9–142)	0.01^*^
	Femoral nerve sensitivity	168 (146–191)	120 (96.1–144)	0.01^*^
Duration of motor blockade (min)	Return of patellar reflex	117 (91.3–143)	110 (89.3–131)	0.7
	Return of tibial reflex	129 (104–155)	109 (92.7–125)	0.2
	Proprioception	167 (124–210)	149 (128–171)	0.4
	Ability to walk	189 (160–219)	216 (160–273)	0.4
Joint palpation (min)		152 (122–182)	117 (100–134)	0.06

Local block outcomes (sensory blockade, motor blockade, and pain) were recorded at 30, 60, 120, 180, and 240 min after extubation in the two study groups. Data are presented as mean (95% confidence interval); *n* = 10 for all groups.

^*^A *p*-value < 0.05 was considered statistically significant compared to group C.

**Table 5:** Physiological variables recorded at 60 (T60), 120 (T120), 180 (T180), and 240 (T240) min after extubation in the two study groups.

	**Group**	**T 60**	**T 120**	**T 180**	**T 240**
HR (beats per minute)	D	97 ± 26	95 ± 28	85 ± 36	98 ± 23
	C	108 ± 15	118 ± 18	111 ± 10	93 ± 26
RR (breaths per minute)	D	21 ± 5	20 ± 3	20 ± 4	23 ± 5
	C	21 ± 3	18 ± 3	21 ± 6	22 ± 10
T (°C)	D	37.2 ± 0.5	38.1 ± 1	38.5 ± 0.7	38.5 ± 0.6
	C	37 ± 0.4	38 ± 0.2	38.5 ± 0.3	38.6 ± 0.2

Data are presented as mean ± standard deviation.

HR, heart rate; RR, respiratory rate; T, temperature.

**Figure 2:**
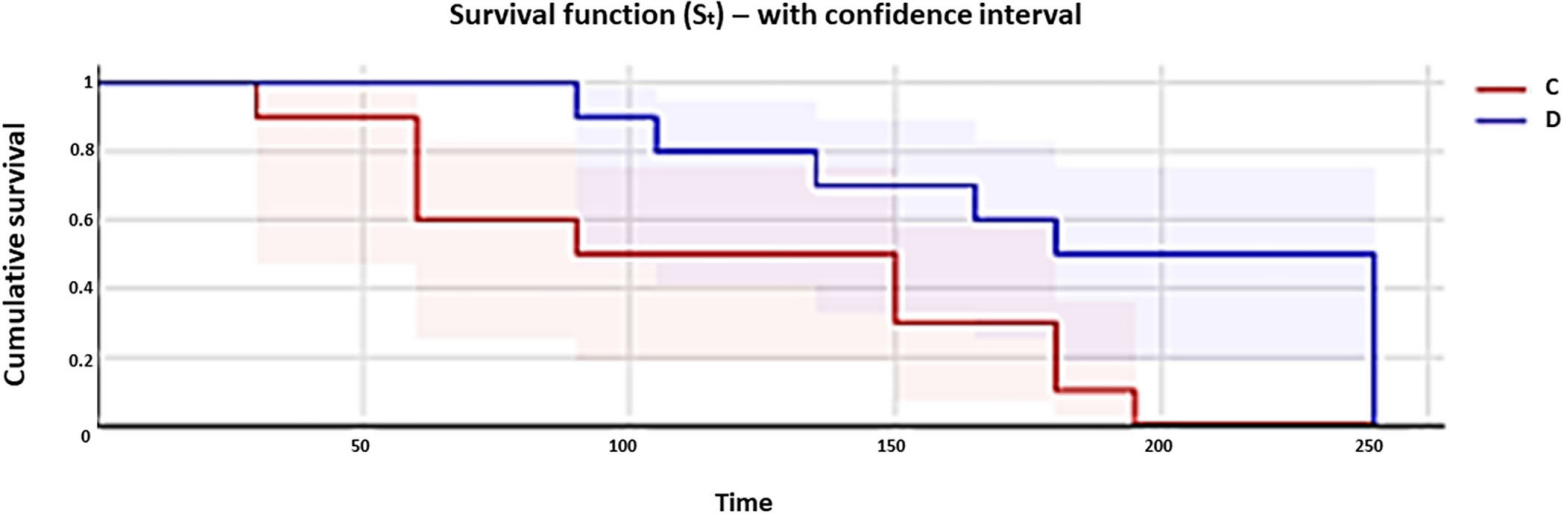
Kaplan–Meier curves show the percentage of rescue analgesia requirement (0.2 mg/kg of methadone administered intramuscularly with 1 mg/kg of subcutaneous robenacoxib) in dogs of group C (red line) and group D (blue line) during the 4 h after extubation. Curves are reported in comparison.

The overall incidences of postoperative rescue analgesia in groups D and C were 5/10 (50%) and 10/10 (100%) cases, respectively (*p* = 0.019); the Kaplan–Meier curve is shown in Figure 2. The comparison of curves regarding the percentage of rescue analgesia requirement over time was statistically different between the groups (log-rank test, *p* = 0.033). Median times at which 50% of the dogs required analgesia were 210 (95% CI: 135–240) and 120 (95% CI: 60–195) min in groups D and C, respectively (Figure 2).

## 4. Discussion

This study's results confirm the hypothesis that the concomitant CRI of dexmedetomidine at a dose of 1 μg/kg/h and the peripheral administration of lidocaine for sciatic-femoral nerve block in dogs undergoing knee surgery can prolong the sensory, without prolonging the motor, blockade. There was ~40% prolongation of the sensory block in group D compared to group C and a significant reduction in opioid administration during the postoperative period.

Dexmedetomidine is commonly used in small animal anesthesia because of its potent sedative and analgesic properties. Its use as a CRI during surgery is considered part of the protocol of partial or total intravenous anesthesia owing to its capacity to balance the anesthetic plane and improve analgesia and recovery (24–26). Its use at low dosages and without a bolus has been proven to mitigate hemodynamic side effects (25).

Herein, we demonstrated that a dexmedetomidine infusion prolongs the duration of the sensory blockade of perineural lidocaine by ~40 min, without any impact on motor function. The adjuvant action of dexmedetomidine in RA is well-recognized in the literature, and its use has been proven to be more effective in prolonging the sensory block, under local administration rather under systemic administration (27). Numerous mechanisms and sites of action, including supraspinal, spinal, and peripheral, have been hypothesized to explain the adjuvant effect of alpha2-agonists on LAs. Theories have been proposed to explain the alpha2-agonist drug's supraspinal action, including the binding of the alpha2-receptor at the level of the locus coeruleus in the brainstem, which decreases the release of norepinephrine and inhibits sympathetic activity (28), and the action of the drug at the level of the dorsal horn, which changes the modulation of the nociceptive impulse (9). At the peripheral level, its function was shown to depend on an inhibitory role in delayed rectifier K+ and Na+ currents, reducing neuronal activity. Furthermore, dexmedetomidine has been reported to prolong nerve blocks by blocking the hyperpolarization-activated cation current, which is considered the principal peripheral mechanism of action of dexmedetomidine (14, 16, 29), and is more pronounced in C fibers (pain) than in A fibers (motor). Its adjuvant effect should be related to vasoconstriction mediated by the alpha2-agonist at the injection site, which could reduce the systemic absorption of the LA and prolong its local duration of action (30). Considering the data published by Acquafredda et al., the adjuvant effect of intramuscular injection of dexmedetomidine was lower than that produced by the local administration with lidocaine. This finding may suggest that the systemic effects of dexmedetomidine on block duration would be due to the supraspinal action of the drug rather than a local effect mediated by vasoconstriction, especially at the low dose used in the mentioned study.

Considering that the anesthetic protocol used in the present study did not include systemic analgesic drugs, such as opioids and anti-inflammatory drugs, the efficacy of the dexmedetomidine infusion on perioperative analgesia was also confirmed by the significant reduction in rescue analgesia administered in group D during the postoperative observational period. Dogs that received the dexmedetomidine infusion in group D required less analgesia in the postoperative period than dogs in group C.

In the current study, the use of a low-dose infusion of dexmedetomidine without a loading dose produced minimal cardiovascular side effects, as demonstrated by the very low incidence of hypotension in both groups and the absence of bradycardia. This finding is in line with the results of a previous study (26) that also proved that a dexmedetomidine infusion prolonged the recovery time in terms of the return of head movements and sternal recumbency after extubation.

Lidocaine was chosen as the LA for this investigation because it acts more quickly and has a shorter duration than bupivacaine or ropivacaine in dogs (31, 32). Thus, it should be considered an excellent choice for examining the additive analgesic effects of co-administered medications because of its fast offset on nerve fibers when compared to other LAs (31). Clearly, evaluation of a dexmedetomidine infusion in combination with longer-lasting LAs is desirable for future animal studies.

The limitations of this study are, first, that the time of the dexmedetomidine infusion was not standardized between dogs because of the surgery time; thus, the total amount of drug administered was not exactly the same between dogs. Second, the short postoperative monitoring time (4 h) did not permit a complete picture of the 24-h effects of this drug, as reported in other similar studies (27).

## 5. Conclusions

Dexmedetomidine administered as an intravenous CRI (1 μg/kg/h) combined with local lidocaine increased the duration of the sensory sciatic and femoral nerve blocks and reduced the requirement for additional analgesia during the immediate postoperative period. Further studies are required to establish the efficacy of dexmedetomidine CRI in combination with longer-lasting LAs, such as ropivacaine or bupivacaine.

## Data availability statement

The raw data supporting the conclusions of this article will be made available by the authors, without undue reservation.

## Ethics statement

This study was approved by the Ethical Committee for Veterinary Clinical and Zootechnical Studies of the Department of Emergency and Organ Transplantation (D.E.O.T.) at the University of Bari, Italy (certificate of approval number: 02/2021). Informed consent was obtained from all the owners of the dogs before they were included in the study. This study followed the Consolidated Standards of Reporting Studies Statement 2010 for presenting randomized clinical trials. Written informed consent was obtained from the owners for the participation of their animals in this study.

## Author contributions

MS, FS, and LL: study design and data analysis. MS, CA, AS, AC, and FS: study execution. MS: writing the manuscript. LL, CA, AS, AC, and FS: manuscript review. All authors have contributed to the manuscript and approved the submitted version.
